# Lymphocyte Subpopulation Number and Function in Infancy

**DOI:** 10.1155/1992/64292

**Published:** 1992

**Authors:** Debra A. Stern, Mary Jane Hicks, Fernando D. Martinez, Catharine J. Holberg, Anne L. Wright, Jacob Pinnas, Marilyn Halonen, Lynn M. Taussig

**Affiliations:** 1 Respiratory Sciences Center and the Departments of Pathology, Internal Medicine, Pharmacology, and Pediatrics University of Arizona Health Sciences Center Tucson Arizona 85724 USA; 2 Respiratory Sciences Center University of Arizona HSC Tucson Arizona 85724 USA

**Keywords:** Normal values, cellular immunology, infancy, lymphocytes

## Abstract

Normal values for percentages of lymphocyte subpopulations and functional responses to mitogen stimulation in infancy are not well established. In the present study, lymphocyte subpopulations were examined in umbilical cord blood samples and in peripheral blood samples drawn before 7 and 24 months of age (mean age 10.4 months) from a healthy population of infants born in Tucson, Arizona. Results indicate significant increases occurred from birth to later infancy in the percentages of total T cells (CD3), T-cell subsets (CD4, CD8) and B cells (CD20). The CD4/CD8 ratio and the functional responses to ConA and PWM mitogens significantly decreased from birth to later infancy. PHA responsiveness did not show a significant change. Results from cross-sectional analyses (*n*=271) were supported in a smaller longitudinal subset (*n*=37). There were no detectable ethnic- or gender-related differences in cord blood or samples obtained in later infancy. The normal values established in this study will be useful in studies of immune-system maturation and in the clinical evaluation of newborns, infants, and toddlers suspected of either acquired or congenital immune-deficiency states.


*Developmental Immunology*, 1992, Vol. 2, pp. 175–179
Reprints available directly from the publisher
Photocopying permitted by license only

© 1992 Harwood Academic Publishers GmbH
Printed in the United Kingdom

### Lymphocyte Subpopulation Number and Function in Infancy

DEBRA A. STERN,*† MARY JANE HICKS,† FERNANDO D. MARTINEZ,† CATHARINE J. HOLBERG,† ANNE L. WRIGHT,† JACOB PINNAS,† MARILYN HALONEN,† and LYNN M. TAUSSIG†

†*Respiratory Sciences Center and the Departments of Pathology, Internal Medicine, Pharmacology, and Pediatrics, University of Arizona Health Sciences Center, Tucson, Arizona 85724*

Normal values for percentages of lymphocyte subpopulations and functional responses to mitogen stimulation in infancy are not well established. In the present study, lymphocyte subpopulations were examined in umbilical cord blood samples and in peripheral blood samples drawn before 7 and 24 months of age (mean age 10.4 months) from a healthy population of infants born in Tucson, Arizona. Results indicate significant increases occurred from birth to later infancy in the percentages of total T cells (CD3), T-cell subsets (CD4, CD8) and B cells (CD20). The CD4/CD8 ratio and the functional responses to ConA and PWM mitogens significantly decreased from birth to later infancy. PHA responsiveness did not show a significant change. Results from cross-sectional analyses ($n$=271) were supported in a smaller longitudinal subset ($n$=37). There were no detectable ethnic- or gender-related differences in cord blood or samples obtained in later infancy. The normal values established in this study will be useful in studies of immune-system maturation and in the clinical evaluation of newborns, infants, and toddlers suspected of either acquired or congenital immune-deficiency states.

KEYWORDS: Normal values, cellular immunology, infancy, lymphocytes.

### INTRODUCTION

Relatively few studies have investigated lymphocyte subpopulations in umbilical cord blood and/or in peripheral blood during the first 2 years of life. Of these, most included small sample sizes or did not investigate the relationship between values at birth and longitudinal follow-up (Christiansen et al., 1976; Andersson et al., 1981; Hicks et al., 1983a, 1983b; Pahwa et al., 1985; Tainio, 1985; Thomas and Linch, 1983). Documentation of the maturation of the immune system is needed as a basis for further studies examining age-related susceptibility to infectious agents. The establishment of accurate age-related normal values would also allow more meaningful immunologic evaluation, at birth and in the neonatal period, of infants born to HIV seropositive mothers (Blanche et al., 1989; Rogers et al., 1989) and of infants suspected of having a congenital immune deficiency.

This study examines lymphocyte subpopulations in healthy infants at birth and within the first 2 years of life. The present study employs tests of both lymphocyte numbers and function, large sample sizes, and (in a subset of the population) longitudinal data for birth and one subsequent sample.

### MATERIALS AND METHODS

### Study Subjects

Infants in this study were born between 1981 and 1984, to parents who were members of a HMO in Tucson, Arizona. A portion of these infants were enrolled into the Tucson Children's Respiratory Study (CRS), a longitudinal prospective study of the risk factors for the development of acute and chronic respiratory disease. Selection criteria and

*Corresponding author. Present address: Respiratory Sciences Center, University of Arizona HSC, Tucson, Arizona 85724.

characteristics of this healthy population have been reported (Taussig et al., 1989). Additionally, cord blood samples collected from infants born during the same time period (1981–1984) but not enrolled in the CRS were included in the present study. All participants signed human subject consent forms approved by the College of Medicine Human Subjects Committee.

Detailed health information and the opportunity to analyze peripheral blood samples collected during later infancy were available for the infants enrolled in the CRS. Therefore, CRS enrolled and nonenrolled infants were initially separated to determine comparability with the CRS enrolled infants referred to as group A and the nonenrolled infants as group B.

Lymphocyte studies were performed on specimens collected from 271 infants at birth and/or at the "well baby check" at either 7≤9, >9≤12, or >12≤24 months of age. Forty-one of the group A infants had only a cord blood specimen, 37 had both cord blood and a longitudinal sample drawn between 7 and 24 months, and 102 had no cord blood sample, only the specimen drawn between 7 and 24 months. Cord blood specimens were obtained from all 91 group B infants.

The number (%) of group A infants in each age group tested between 7–24 months of age (mean age 10.4 months, SD=2.6) were as follows: 29 (21%) were tested between 7 and 9 months (mean age 8.5 months); 81 (58%) were tested between >9 and 12 months (mean age 9.8 months); and 29 (21%) were tested between >12 and 24 months (mean age 14.4 months).

Group A infants were assigned to one of two ethnic categories based on maternal self-report; there were 32 Hispanic infants (both Hispanic parents) and 148 infants with one or both Anglo parents. The remaining 7 group A infants were of other ethnic backgrounds. Ethnicity information was not available for group B infants.

### Laboratory Materials and Methods

Methods of blood collection and laboratory procedures have been previously described (Hicks et al., 1981; Kibler et al., 1985, 1986). Briefly, blood was collected in heparin and lymphocyte subset phenotyping performed on lymphocyte suspensions prepared by a standard density gradient centrifugation technique (Hicks et al., 1981). Indirect immunofluorescence microscopy using monoclonal antibodies for measuring total T cells [OKT3 (CD3)], T helper cells [OKT4 (CD4)], T suppressor/cytotoxic cells [OKT8 (CD8)] (Ortho Diagnostics, Raritan, NJ), and B cells [B1 (CD20); Coulter Immunology, Hialeah, FL] was used as described (Kibler et al., 1985, 1986). Smears were examined with a Zeiss fluorescent microscope equipped to give epiillumination.

The lymphocyte stimulation method used optimal doses of phytohemagglutinin (PHA, GIBCO, Grand Island, NY), pokeweed mitogen (PWM, GIBCO), and concanavalin A (ConA, Calbiochem, San Diego, CA) as previously reported (Hicks et al., 1983a; Kibler et al., 1986). Values are reported as mean counts per minute (cpm) for stimulated wells.

### Statistical Analysis

Analyses were performed using the Statistical Package for the Social Sciences, version 4.0, on a SUN 4 computer system. All variables were determined to be normally distributed and parametric analyses performed. The lower 2.5 and upper 97.5 percentiles as determined directly from each frequency distribution were used as measures of lower and upper limits of normal values (Elveback et al., 1970). Paired t-tests were used to test for differences between the cord blood and 10-month samples. Unpaired samples were analyzed exclusive of the paired samples and unpaired t-tests used to determine significance. Analysis of variance was used to identify differences among the age group means and for gender- and ethnic-specific groups. A two-tailed $p$ value of less than 0.05 was considered significant for all tests.

### RESULTS

Males and females were distributed similarly in groups A and B, with 53% of group A and 55% of group B male. For the cord blood samples, the mean of each immunologic variable for group A and group B were compared. No significant differences were observed except for the lymphocyte responses to Concanavalin A (ConA) and pokeweed (PWM) mitogens. The mean response for the group A infants was slightly higher than the group B infants; values in cpm$\times 10^3$ for ConA mean±SE group A: 89±5, $n$=59; group B: 73±5,

TABLE 1
Mean Values for Cellular Immune Variables in Cord Blood and 10-Month Samples

| Cellular immune variables | $n^a$ | Cord blood mean±SD (2.5/97.5)[b] | $n^c$ | 10-month mean±SD (2.5/97.5)[b] |
|---|---|---|---|---|
| CD3 (%) | 152 | 64±11 (42–82) | 127 | 72±8[d] (55–85) |
| CD4 (%) | 167 | 48±10 (25–67) | 133 | 51±8[e] (32–64) |
| CD8 (%) | 167 | 16±7 (6–30) | 132 | 19±6[e] (9–31) |
| CD4/CD8 ratio | 166 | 3.5±1.5 (1–6) | 131 | 3.0±0.9[e] (2–5) |
| B cells (%) | 161 | 15±5 (4–24) | 126 | 17±5[e] (5–23) |
| PHA (cpm×$10^3$) | 131 | 89±56 (3–202) | 64 | 79±51[f] (5–193) |
| ConA (cpm×$10^3$) | 124 | 81±41 (2–155) | 64 | 46±28[d] (11–113) |
| PWM (cpm×$10^3$) | 124 | 25±17 (2–64) | 64 | 11±11[d] (0.3–37) |

[a]Values from group A and group B were pooled and an overall mean value for both groups given. Group B infants had significantly lower responses to ConA and PWM mitogen (see Results).
[b]Determinations of the lower 2.5 and upper 97.5 percentiles of the frequency distribution.
[c]Ten-month values represent an overall mean of values obtained from the 7≤9, >9 ≤12, and >12≤24 month age groups. There were no significant differences in mean values between these age groups.
[d]$p<0.001$.
[e]$p<0.05$.
[f]NS $p$ values for unpaired t-test of cord and 10-month samples.

TABLE 2
Longitudinal Change in Cellular Immune Variables from the Cord Blood Sample to the 10-Month Sample

| Cellular immune variables | $n$ | Difference[a] mean (SD) | $p$ value[b] |
|---|---|---|---|
| CD3 (%) | 36 | +10 (11) | 0.000 |
| CD4 (%) | 36 | +6 (12) | 0.005 |
| CD8 (%) | 35 | +5 (8) | 0.001 |
| CD4/CD8 ratio | 35 | −0.6 (1.4) | 0.012 |
| B cells (%) | 34 | +3 (6) | 0.005 |
| PHA (cpm×$10^3$) | 18 | +17 (75) | 0.352 |
| ConA (cpm×$10^3$) | 18 | −40 (50) | 0.003 |
| PWM (cpm×$10^3$) | 18 | −18 (17) | 0.000 |

[a]The difference is the 10-month mean value minus the cord mean value.

[b]$p$ value for the change from the cord blood sample to the 10-month sample was determined by a paired t-test.

$n$=65, $p$=0.03; for PWM mean±SE group A: 28±2, $n$=59; group B: 22±2, $n$=65, $p$=0.04.

The means and lower 2.5 and upper 97.5 percentiles for each of the immunologic variables (%CD3, %CD4, %CD8, %B cells, CD4/CD8 ratio, PHA, ConA, and PWM mitogens) in cord blood and the later infancy samples are reported in Table 1. Group A and group B are combined for the cord blood values. No significant differences in mean values were detectable between the 7≤9, >9≤12, and >12≤24 month age groups for any of the immunologic variables. These age groups were therefore pooled and designated the "10-month" sample.

Cord blood and 10-month samples were analyzed separately for the infants with paired and unpaired samples. For the paired samples, the percentage of CD3, CD4, CD8, and B cells all increased significantly from birth to the follow-up sample (Table 2). The CD4/CD8 ratio decreased significantly as well as the lymphocyte functional responses to ConA and PWM mitogens. No significant change was observed for the functional response to PHA. Unpaired samples demonstrated changes similar in direction and significance to those observed in the paired samples (Table 1).

Analysis of variance indicated no significant gender- or ethnic-related effects on any of the immunologic variables for the cord blood or 10-month samples.

### DISCUSSION

The primary findings of this study include a significant increase in percentages of total T cells (CD3), T-cell subsets (CD4 and CD8), and B cells (CD20) from birth to later infancy (mean age 10.4 months); and significant decreases in the CD4/CD8 ratio and in the functional responsiveness to ConA and PWM mitogens. The implication that the cross-sectional changes were age-related was confirmed by similar longitudinal changes in a subset of infants with matched samples.

In a previous study, we reported mean values for cord blood that were very similar to the present values for all variables (Kibler et al., 1986). The methods were similar to the present study though the sample size was smaller. Thomas and Linch (1983) demonstrated a CD4/CD8 ratio in 1-week-old infants of 2.8. Our cord blood CD4/CD8 ratio (3.5) was higher than this neonatal value, which suggests that the CD4/CD8 ratio may decrease immediately after birth. In a sample of 17 healthy infants less than 1 week old, Pahwa et al. (1985) reported mean percentages of CD3, CD4, and CD8 cells very similar to our values for cord blood (63%, 45%, and 20%, respectively), even though different methodologies were employed.

Mean percentages of CD3 and CD4 positive lymphocytes (62% and 40%, respectively) derived from data displayed by Tainio (1986) for infants at about age 10 months were lower than the present study (71% and 51%, respectively). The percentage of CD8 and B cells in their study (21% and 16%, respectively) were very similar to the present mean values (19% and 17%, respectively). Differences may be due to the smaller sample size in the Tainio study ($n$=22–24, paired samples $n$=6–13).

Comparison of the 10-month values reported here to adult values reported previously by Kibler et al. (1986), whose methods are similar to the present study, did not reveal any differences in the percentages of CD3, CD4, B cells, and proliferative responses to PHA and PWM mitogens. However, the percentage of CD8 cells was lower in the 10-month samples compared to adults resulting in a higher 10-month CD4/CD8 ratio. The proliferative response to ConA was higher in 10-month samples compared to adults (Kibler et al., 1986). Thus, maturational changes in values for CD3, CD4, B cells, and proliferative responses to PHA and PWM mitogens after birth appeared to have occurred by 10 months of age, because the 10-month values were similar to adult values.

The apparent increased responsiveness to mitogens in cord blood may be associated with the *in vivo* state of activation of lymphocytes. It has been observed that spontaneous DNA synthesis was higher in cord blood (Campbell et al., 1974). Prindull et al. (1975) also observed higher numbers of spontaneously labeling cells in cord blood compared to adult samples.

No ethnic- or gender-related differences were demonstrated in the present study. Kibler et al. (1986) did not find any differences in these mean values between boys and girls in a subset of individuals included in the present study. Thomas and Linch (1983) also did not find any differences in the absolute number of lymphocytes or subsets between boys and girls, when sampled neonatally.

Values for the mean percentages of CD3, CD4, and CD8 (7.3%, 2.9%, and 6.1%, respectively) reported for nine infants aged 1–18 months with severe combined immunodeficiency (Davies et al., 1983) were below the lower 2.5 percentiles for the estimate of the population mean in our 10-month samples. This lends credence to the contribution of the present study in providing upper and lower limits of normal for these parameters.

In summary, the results of this study indicated that different reference ranges should be used to evaluate the immune system at birth compared to 10 months of age. The normal values established in this study will be useful for comparison of infants with infectious illnesses and in the clinical evaluation of newborns, infants, and toddlers suspected to have acquired or congenital immune deficiency states.

### ACKNOWLEDGMENTS

This work was supported by NIH SCOR grant (HL14136).

*(Received September 6, 1991)*

*(Accepted September 17, 1991)*

### REFERENCES

Andersson U., Bird A.G., Britton S., and Palacios R. (1981). Humoral and cellular immunity in humans studied at the cell level from birth to two years of age. Immunol. Rev. **57:** 5–37.

Blanche S., Rouzioux C., Moscato M.L.G., et al. (1989). A prospective study of infants born to women seropositive for human immunodeficiency virus type I. New Eng. J. Med. **320:** 1643–1648.

Campbell A.C., Waller C., Wood J., Aynsley-Green A., and Yu V. (1974). Lymphocyte subpopulations in the blood of newborn infants. Clin. Exp. Immunol. **18:** 469–482.

Christiansen J.S., Osther K., Peitersen B., and Bach-Mortensen N. (1976). B, T and null lymphocytes in newborn infants and their mothers. Acta Paediatr. Scand. **65:** 425–428.

Davies E.G., Levinsky R.J., Butler M., Thomas R.M., and Linch D.C. (1983). Lymphocyte subpopulations in primary immunodeficiency disorders. Arch. Dis. Child. **58:** 346–351.

Elveback L.R., Guillier C.L., and Keating F.R. (1970). Health, normality, and the ghost of Gauss. J.A.M.A. **211:** 69–75.

Hicks M.J., Jones J.F., Thies A.C., and Minnich L.L. (1981). The effect of lymphocyte recovery on lymphocyte typing results. Amer. J. Clin. Pathol. **76:** 745–752.

Hicks M.J., Jones J.F., Thies A.C., Weigle K.A., and Minnich L.L. (1983a). Age-related changes in mitogen-induced lymphocyte function from birth to old age. Amer. J. Clin. Pathol. **80:** 159–163.

Hicks M.J., Jones J.F., Minnich L.L., Weigle K.A., Thies A.C., and Layton J.M. (1983b). Age-related changes in T- and B-lymphocyte subpopulations in the peripheral blood. Arch. Pathol. Lab. Med. **107:** 518–523.

Kibler R., Lucas D.O., Hicks M.J., Paulos B.T., and Jones J.F. (1985). Immune function in chronic active Epstein-Barr virus infection. J. Clin. Immunol. **5:** 46–54.

Kibler R., Hicks M.J., Wright A.L., and Taussig L.M. (1986). A comparative analysis of cord blood adult lymphocytes: interleukin-2 and interferon production, natural killer cell activity, and lymphocyte populations. Diag. Immunol. **4:** 201–208.

Pahwa S., Sia C., Harper R., and Pahwa R. (1985). T lymphocyte subpopulations in high-risk infants: Influence of age and blood transfusions. Pediatrics **76:** 914–917.

Prindull G., Prindull B., Ron A., and Yoffey J.M. (1975). Cells in spontaneous DNA synthesis in cord blood of premature and full-term newborn infants: An autoradiographic study. J. Pediatr. **86:** 773–778.

Rogers M.F., Ou C.Y., Rayfield M., et al. (1989). Use of the polymerase chain reaction for early detection of the proviral sequences of human immunodeficiency virus in infants born to seropositive mothers. New Eng. J. Med. **320:** 1649–1654.

Tainio V.M. (1985). Lymphocyte subsets in infants: Relationship to feeding, atopy, atopic heredity and infections. Int. Arch. Allergy Appl. Immunol. **78:** 305–310.

Taussig L.M., Wright A.L., Morgan W.J., Harrison H.R., Ray C.G., and The Group Health Medical Associates (1989). The Children's Respiratory Study I. Design and implementation of a prospective study of acute and chronic respiratory illness in children. Amer. J. Epidemiol. **129:** 1219–1231.

Thomas R.M., and Linch D.C. (1983). Identification of lymphocyte subsets in the newborn using a variety of monoclonal antibodies. Arch. Dis. Child. **58:** 34–38.

